# An interview with David H Farb, Section Editor for Basic Pharmacology

**DOI:** 10.1186/2050-6511-14-42

**Published:** 2013-08-30

**Authors:** David H Farb

**Affiliations:** 1 Department of Pharmacology and Experimental Therapeutics, Laboratory of Molecular Neurobiology, Boston University School of Medicine, Boston, MA, 02118, USA

## 

David H Farb is Professor and Chair of the Department of Pharmacology and Experimental Therapeutics at Boston University School of Medicine. Dr. Farb is Principal Investigator and Director of the Laboratory of Molecular Neurobiology and Director of the university-wide NIGMS training program in Biomolecular Pharmacology. A renowned neuropharmacologist, his research integrates existing electrophysiological, behavioral, pharmacological, and molecular genetics technologies in a novel systems-level platform to establish a molecular dissection of spatial memory. In this interview we find out a little more about the key issues in this field of research.

## Could you tell us about the career path that led you to your current position and research interests?

I suppose anxiety was one of the early motivators in my career; more specifically, the study of the role of GABA_A_ receptors in the development of anxiety disorders, ranging from generalized anxiety to post-traumatic stress. As a post-doctoral fellow, I worked with then MD/PhD student Dennis W. Choi and Gerald D. Fischbach in the Department of Pharmacology at Harvard Medical School to uncover the cellular mechanisms of action of benzodiazepines-the class of agents that serve not only as anxiolytics, but sedative hypnotics and anticonvulsants as well. We showed that benzodiazepines act by positive allosteric modulation of GABA_A_ receptors [1], and we provided the first measurement of the synthesis, degradation and turnover of a neurotransmitter receptor in the central nervous system [2]. My research today continues to be primarily focused on the discovery and development of neuromodulators as therapeutic agents, and on the structure, function, and cellular dynamics of ion channels and receptors in the brain and spinal cord [3]. For example, we have shown that the endogenous neuroactive steroid pregnenolone sulfate, which is formed from cholesterol via sulfotransferase action on pregnenolone, creates a branch point for activation of an allosteric modulator of NMDA and non-NMDA glutamate receptor function [4]. We have recently reported that this unique sulfated steroid can stimulate the trafficking of functional NMDA receptors to the cell surface via a non-canonical G-protein and Ca^++^-dependent mechanism, a finding that may further the development of treatments not only for schizophrenia, but also for other conditions associated with malfunctioning NMDA receptors, such as age-related decreases in memory and learning ability [5].

## What are your aspirations for the ‘Basic Pharmacology’ section of *BMC Pharmacology and Toxicology*?

In recent years, open access journals have become extremely influential in shaping the exchange of scientific knowledge, and *BMC Pharmacology and Toxicology* has been in the forefront of this educational endeavor. In particular, the journal has played a major role in facilitating the timely publication of new scientific research, creating an open forum for discussion of current research trends, and enabling a versatile platform for publication of research findings. My aspirations for the ‘Basic Pharmacology’ section are to continue this timely direction of the journal, publishing translational content that reflects emergent trends in biomedical research and bridges the fields of pharmacology, clinical pharmacology and toxicology. To this end, we encourage submission of research reports in all therapeutic areas broadly encompassing pharmacology and signaling, with incorporation of basic science drug discovery and translational medicine. We are also enthusiastic in our goal to disseminate research that incorporates existing and rapidly evolving leading edge technologies to bridge systems-level inquiries with those at the cellular and molecular levels, as well as research such as computational modeling of systems based on technology platforms that can lead to a greater understanding of the genetic basis of disease [6].

We are also striving to balance two competing, but not diametrically opposed, forces in scientific publishing: “prestige” science versus less trendy but still exciting and, ideally, solid science. Clearly, citation rates are a useful indicator for measuring the interest given to a publication over a meaningful period of time. It is important to recognize, however, that the growing emphasis on impact factors in scientific publishing is problematic [7,8], and trendiness rather than reproducibility is being misused by some to guide merit ratings awarded by NIH and other funding agency review panels and may be a driver of the disturbingly high rate of failure, more than 50%, to reproduce findings reported in some high impact journals. Scientists at Amgen reported that they could reproduce the key results of only a small fraction (11%) of 53 publications (many in high impact journals) they attempted to validate [9]. As communicators of factual information, it is our responsibility to look through the prism of time, trying ever to focus on the progress of science. For this reason, the open access format of *BMC Pharmacology and Toxicology* may serve to counterbalance the pressures on scientists by focusing more on solid science. We intend to be nimble, staying on the leading edge of translational and basic research through a policy of openness to new ideas that are supported by convincing, reproducible evidence.

## Which areas of research would you be particularly interested in seeing submitted to your section?

It’s about publishing all of the science that’s fit to digitize, to spin a phrase. I believe our readership would benefit from research that explores the increasingly pivotal role of personalized medicine in the development of more precise therapeutic approaches to a range of diseases. Genome-wide association studies have enabled a greater understanding of individual differences in disease susceptibility, age of onset, and sexual differentiation in the response to treatment, and greatly underscore the need for an expansion of these types of approaches. For example, it would be of interest to assess how, or even whether, antiviral therapeutics differentially affect the responsiveness of female patients to treatment for sexually transmitted diseases. I am particularly interested in seeing research that embraces new technologies to facilitate in-depth scientific inquiry in any area of pharmacological sciences.

## What challenges currently exist in the field of pharmacology?

Despite intense drug discovery efforts, disorders of the central nervous system have suffered the most from a lack of newly developed therapeutics. Target-based drug discovery, or the process of identifying a drug candidate that acts at a validated target molecule, is the most widely used approach. Although this approach has been successful in identifying drugs for previously validated targets, it has been less successful when novel targets are involved. This is especially true in neuropsychiatric disorders with multifaceted etiologies, and we should consider that a systems-level approach will be needed to model these disease states. Renewed efforts to identify allosteric modulators of multiple receptor types (G protein-coupled receptors in particular) are expected to yield rich dividends in the near future [10]. Several of these compounds have progressed to clinical trials. One promising therapeutic approach has been the development of allosteric modulators, which target a site that is distinct from orthosteric agonist binding. Allosteric positive modulators targeting the benzodiazepine binding site of GABA_A_ receptors are a classic example of early successes utilizing this approach [1,11]. These compounds are prescribed frequently for the treatment of anxiety disorders, epilepsy, and insomnia, as well as pre-anesthetic sedation and muscle relaxation. Although recent advances in understanding receptor subtype pharmacology in rodent models hold great promise for understanding the biology of drug action, the science of translation to humans remains a conundrum, as evidenced by the failure of research in mouse transgenic models to form a platform for the translation of pharmacological effects in mouse models into anxiolytics for use in human [12]. Breaking the barrier of translational sciences is the single greatest challenge for modern pharmacology, as a whole new pharmacopeia awaits discovery!

Another key challenge in pharmacology is optimizing the use of the ever-increasing amount of genetic information available to us. While the Human Genome Project was rightly heralded as a milestone by those who referred to it as a new platform for aiding medical research, it is nonetheless misleading to think of it as a “roadmap” as we normally understand the term. The genotype may identify a particular phenotype, but does not guide us in one direction or another therapeutically, at least at this time; rather, it provides clues to a complex roadway of potential therapeutic targets [13]. In this respect, *BMC Pharmacology and Toxicology* can provide a useful venue for communicating broad-ranging advances that bridge molecular identities with clinical endpoints without having to assert claims to having solved the problem. We welcome the excitement of the discovery process!

## How might these challenges be met by future research efforts?

Endeavors to develop allosteric modulators are complemented by drug discovery efforts incorporating a multitude of innovative approaches. A prime example is the use of designer receptors to aid in deciphering the involvement of specific receptors and transmitters in disease [14]. The close scrutiny of protein-protein interactions, combined with innovative methods of generating protein structure-based molecular inhibitors targeting specific subsets of receptors and intracellular signaling moieties, holds promise for generating novel molecular probes that are useful both as research tools and as future therapeutics. A greater emphasis on structure-based drug discovery studies aided by computational and structural modeling efforts bodes well for future success. In fact, computational and structural modeling studies are of great importance not only in drug design, but also in understanding the mechanistic underpinnings of various disorders ranging from autism and post-traumatic stress disorder to angina and hypertension.

## What recent advances in the field of pharmacology have particularly interested you?

There are several fields that I think are of particular promise moving forward, including molecular genetics, nanomedicine, optogenetics, tissue engineering, and stem cell technology. To start with genetics as an example, Cre-mediated genetic manipulation in animal models is likely to be of great utility in querying receptor involvement in various disease states and identifying relevant signaling cascade entities. In addition, remarkable progress has been achieved in understanding the significance of microRNAs (miRNA) in the regulation of multiple physiological and pathological conditions. The successful use of antagomirs (a novel class of synthetic oligonucleotides that inhibit miRNAs) in clinical applications for cancer treatment highlights the impact that this class of small RNAs is likely to have on diagnostics and therapeutics in the future [15,16]. Epigenetic studies are increasingly utilized to aid in the identification of novel therapeutic targets in a wide variety of diseases. I also mentioned optogenetics, which affords a means of regulating neuronal activity by expression of bacterial light-sensing molecules in a nucleus of choice in the brain, doing so in a cell- and pathway-specific manner. This novel technology is shedding light on targets for various disorders, while remaining mindful that existing methods may not report responsivity of neurons accurately, yet in the future may itself yield novel options for discovery science to utilize [17]. Recent advances in tissue engineering and stem cell-based therapeutics also offer tremendous potential for treating disease and identifying new drugs. However, this field is not without limitations and challenges. Identifying extracellular entities and molecular remodeling mechanisms that regulate stem cell differentiation is crucial for the treatment or amelioration of multiple diseases and brain disorders. Cellular reprogramming and generation of inducible pluripotent stem cells from adult cell types aids in creating patient-specific stem cells. These disease-relevant cell types provide a novel means of modeling specific diseases and identifying relevant pathways, biomarkers and treatments. Disorders ranging from cardiovascular to psychiatric pathologies such as schizophrenia are benefiting from this approach.

## Where might new technologies assist with advances in the field of pharmacology?

Toxicity is still the major obstacle in drug development, and overcoming this obstacle will require the development of more streamlined *in silico* or *in vitro* toxicology workflows. The importance of biomarkers, both as diagnostics of disease and as indicators of toxicity associated with drug treatments, cannot be over emphasized. In addition, drug delivery continues to be hindered by the inability of targeted therapeutics to cross various cellular barriers, such as the blood-cancer cell cluster barrier or the blood–brain and blood-nerve fiber barriers. New technologies-including nanotechnology/nanomedicine, *in silico* drug screening, and biophysical research on barrier surfaces-are crucial for addressing these limitations to drug delivery. The importance of translational studies in pharmacology is underscored by the federal endorsement of translational studies, as evidenced by the recent launching of a National Center for Advancing Translational Sciences (NCATS) [18] in partnership with eight major pharmaceutical companies [18]. To achieve an all-inclusive or interdisciplinary pharmacological sciences journal has been a challenge for generations – to do this in a meaningful and useful way will require the participation of our readership who will after all support the task of peer review.

## Competing interests

David H Farb is a Section Editor for *BMC Pharmacology and Toxicology*.

## Authors’ contributions

DHF wrote and approved the final text.

## Pre-publication history

The pre-publication history for this paper can be accessed here:

http://www.biomedcentral.com/2050-6511/14/42/prepub
